# Characteristics of Treatment Seeking Finnish Pathological Gamblers: Baseline Data from a Treatment Study

**DOI:** 10.1007/s11469-012-9411-4

**Published:** 2012-11-21

**Authors:** Tuuli Lahti, Jukka Halme, Maiju Pankakoski, David Sinclair, Hannu Alho

**Affiliations:** 1 Department of Mental Health and Substance Abuse Services, National Institute for Health and Welfare, P.O.Box 30, 00271 Helsinki, Finland; 2 Department of Behavioural Sciences and Philosophy, Division of Psychology, Faculty of Social Sciences, University of Turku, Turku, Finland; 3 Research Unit of Substance Abuse Medicine, University of Helsinki, Helsinki, Finland

**Keywords:** SOGS, DSM-IV, Pathological gambling, Gambling

## Abstract

This article describes the socio-demographic characteristics and gambling behavior of 39 pathological gamblers who participated in our treatment study in 2009. The inclusion criteria of the study were: score of five or more on both the South Oaks Gambling Screen (SOGS) and a pathological gambling screen based on the Diagnostic and Statistical Manual of Mental Disorders (DSM-IV). The first 39 patients meeting the inclusion criterion were recruited into the study. The average age of the subjects was 39 years, and 80 % were males. The lag-time between active gambling (at least three times per week) and the onset of a pathological gambling problem was short: within 2 years of active gambling, 62 % of the subjects reported having become pathological gamblers. Our results also indicated certain gender-specific differences in the age at initiation and in the severity of the gambling problem.

Gambling is a globally increasing phenomenon while in Finland gambling is also relatively common: 74 % of Finnish adults (aged 15 years and over) have gambled during the last year (Aho and Turja 2007). Pathological gambling (PG) is characterized in the Diagnostic and Statistical Manual of Mental Disorders (DSM-IV) as an impulse control disorder (APA 1994). PG is comparatively rare, but a seriously impairing condition. As much as 1 % of Finns are estimated to be pathological gamblers (SOGS scores five or more) (Aho and Turja 2007). The symptoms of PG usually start during early adulthood and often co-occur with other mental health problems or substance use disorders (Kessler et al. 2008).

Earlier studies have yielded contradictory evidence about the socio-demographic status of pathological gamblers (PG’s): some studies suggest that the large variations in their socio-demographic status is a reflection of the overall population (Granero et al. 2009), whereas others suggest that unemployment and lower educational level may be more common among PG’s than among the population overall (Petry 2002). Also some gender-specific differences in PG have been found: men seem to begin gambling younger and suffer from PG more often than women (Granero et al. 2009; Crisp et al. 2004; Ladd and Petry 2002; Grant and Kim 2002; Tavares et al. 2001; Potenza et al. 2001; Cunningham-Williams et al. 1998; Goudriaan et al. 2004; Lynch et al. 2004). According to earlier studies, the preferred gambling activities also differ between men and women: men seem to have a tendency to gamble on strategic games such as poker and sports betting, whereas women prefer non-strategic games, such as lotteries (Granero et al. 2009; Grant and Kim 2002).

This paper describes in detail the socio-demographic characteristics, gambling behavior, and gender-specific differences among treatment-seeking PGs that participated in the treatment study in 2009. The treatment study explored the impacts of naltrexone pharmacotherapy and a brief intervention for PG, with treatment outcomes reported elsewhere (Lahti et al. 2010).

## Subjects and Methods

The volunteer participants of this study were recruited by announcements in gambling-related internet sites (www.ray.fi, www.pakkopeli.fi, www.a-klinikka.fi, www.peluuri.fi, www.suomi24.fi, www.paihdelinkki.fi) and by two announcements in a widely distributed, free newspaper (Metro) during the first quarter of 2009. In the announcements, those who experienced gambling as a problem were advised to visit the study’s webpage and complete the Finnish version of the South Oaks Gambling Screen (SOGS) and a DSM-IV-based screen (Lesieur and Blume 1987; APA 1994). SOGS includes 16 questions about problems associated with gambling and is used to measure the pathology of gambling. The total score on the SOGS ranges from 0 to 20 (higher values are indicative of worse psychopathologic states: a value of 5 or more indicates probable pathological gambling). The lifetime version of SOGS was used in the present study. The DSM-IV screen for pathological gambling includes 10 questions relating to persistent and recurrent maladaptive gambling behavior. The total score on the DSM-IV screen ranges from 0 to 10 (higher values are indicative of worse gambling-related problems; a value of 5 or more indicates pathological gambling).

Those respondents who scored five or more on both the SOGS and DSM-IV screens were informed about their possible suitability for the study and given the e-mail address to contact the research team. Those who contacted the research team were invited to a visit with a study doctor, who assessed their suitability for the research. The inclusion criteria were: a score of 5 or more on the SOGS and DSM-IV screens and an ability to use the internet and e-mail. The exclusion criteria were: acute hepatitis, severe liver or kidney dysfunction, suicide risk, severe depression or other untreated mental health problem, participation in other gambling research at the same time, the use of drugs (especially opiates), pregnancy; also excluded were prisoners and people with mental illness or mental retardation. The Ethics Board of the Helsinki & Uusimaa hospital district gave permission for this study (permission # EudraCT # 2008-004102-14 and ethical permission # 259/13/03/00/2008). All participants had to be able to read and understand the patient information sheet and sign the informed consent. All participants were free to cease participation in the study whenever they wanted. The patients were not paid or reimbursed for participating.

The socio-demographic variables of the subjects were based on the following questions: what is your age, gender, relationship status, education, employment status, and residence? In addition to these, we asked if the subjects smoked or if they had incurred debts due to their gambling. To record the lag-times between their first gambling experience, active gambling and pathological gambling, we asked the subjects to report the corresponding ages for these stages. The games gambled and gambling behavior were analyzed from the answers given for the SOGS and DSM-IV screens. We used multivariate linear regression modeling to study the effects of relevant socio-demographic variables on SOGS and DSM-IV scores, and on the initiation age of active gambling. Interactions were not examined due to sample size limitations. Logarithmic transformation was performed to normalize the initiation age distribution. Fisher’s exact test was used to compare gender differences in gambling debts. An alpha level of 0.05 was used for all tests.

## Results

**Fig. 1:**
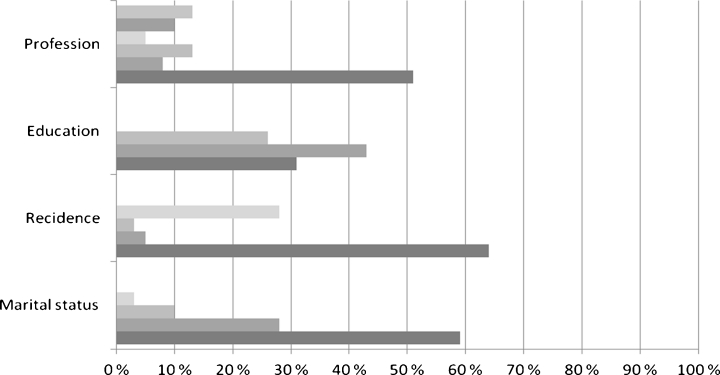
Socio-demographic variables of the subjects

The subjects (*n* = 39) were Finnish adults aged 20–78 years (average age 39 years), with 80 % being male (average age 36 years) and 20 % female (average age 51 years). Most of the subjects were married or cohabited (59 %), lived with their family or with friends (72 %), had at least a secondary education degree (69 %), and were employed (87 %) (Fig. 1). Of the subjects, 49 % were smokers.

According to SOGS, all of the subjects (100 %) had some times gambled more than they had originally planned. The highest amount of money spent on gambling was usually EUR 100–999 per day (44 %) (Table 1). Money loaned for gambling was most often loaned from household funds (69 %) (Table 1). Regression estimates of socio-demographic factors on gambling behavior (SOGS, DSM-IV and initiation age of active gambling) are shown in Table 2. The higher the subject’s score on SOGS, the higher the debts they had incurred due to gambling (β =2.85, se = 1.19, *p* = 0.02). The association between gambling debts and DSM-IV was only of marginal significance (*β* = 0.81, se = 0.47, *p* = 0.10). Men had incurred debts due to gambling significantly more often than women (Fisher’s exact test, *p* = 0.02). Other basic variables (gender, age, education, marital status, residence, or smoking) had no effect on the SOGS or DSM-IV screen scores.

**Table 1:** Money and gambling: highest daily bets and sources of gambling loans

Highest daily bets (% of subjects)	10–99€		100–999€		1000–9999€		>10000	
	5 %		44 %		38 %		13 %	
Sources of loans (% of subjects)	Household funds	Bank/ quick loans	Credit card	Spouse or relatives	Gambling on credit	Private loan givers	Pawnshops or selling property	Selling shares
	69 %	68 %	67 %	56 %	49 %	33 %	31 %	21 %

**Table 2:** Multivariate linear regression model estimates and standard errors

Explanatory variable	Model 1	Model 2	Model 3
	SOGS	DSM-IV	Starting age of active gambling^c^
	β (se)	β (se)	β (se)
(Intercept)	14.77 (2.74)	7.00 (1.13)	3.84 (0.32)
Male gender	−0.57 (1.42)	0.89 (0.57)	−0.56 (0.23)^b^
Age (years)^d^	−0.04 (0.04)	−0.01 (0.01)	-
Elementary school	1.13 (0.97)	0.00 (0.40)	0.02 (0.17)
Married	−1.13 (1.37)	0.13 (0.56)	−0.19 (0.24)
Living alone	−1.61 (1.57)	−0.35 (0.65)	0.11 (0.28)
Smoker	−0.82 (0.93)	−0.01 (0.39)	−0.21 (0.16)
Gambling debts	2.85 (1.19)^b^	0.81 (0.47)^a^	−0.01 (0.20)

^a^ < 0.1

^b^ < 0.05

^c^Logarithmic transformation

^d^There is a self-evident correlation between age and starting age of active gambling, and therefore age is not included as an explanatory variable in Model 3

According to the SOGS results, most of the subjects argued about gambling with their families (95 %) and felt guilt (97 %) due to gambling. Thus most of the subjects 84 % tried to hide their gambling from significant others according to SOGS. The results from the DSM-IV screen also supported this observation. In the DSM-IV screen, 92 % of the subjects reported that they were telling lies to hide their gambling losses, and 74 % had even jeopardized or lost a significant relationship, job, or educational or career opportunity because of gambling. The percentages of responses to the different DSM-IV questions are listed in Table 3.

**Table 3:** Percentage of subjects giving affirmative answers to questions made in DSM-IV. Percentages are presented separately for female (*n* = 8) and male (*n* = 31) populations and for the whole group (*n* = 39)

DSM-IV	Female	Male	All
Thoughts preoccupied with gambling	88 %	100 %	97 %
Need to gamble with increasing amounts of money in order to achieve desired excitement	63 %	81 %	77 %
Repeated unsuccessful efforts to control, cut back, or stop gambling	100 %	97 %	97 %
Restless or irritable when attempting to cut down or stop gambling	88 %	100 %	97 %
Gambling is a way of escaping from problems or of relieving a dysphoric mood	50 %	94 %	85 %
After losing money gambling, often returns another day in order to get even	100 %	100 %	100 %
Lies to family members, therapist, or others to conceal the extent of involvement with gambling	63 %	100 %	92 %
Committed illegal acts, such as forgery, fraud, theft, or embezzlement, in order to finance gambling	0 %	10 %	8 %
Jeopardized or lost a significant relationship, job, or educational or career opportunity because of gambling	25 %	87 %	74 %
Relies on others to provide money to relieve a desperate financial situation caused by gambling	50 %	77 %	72 %

**Fig. 2:**
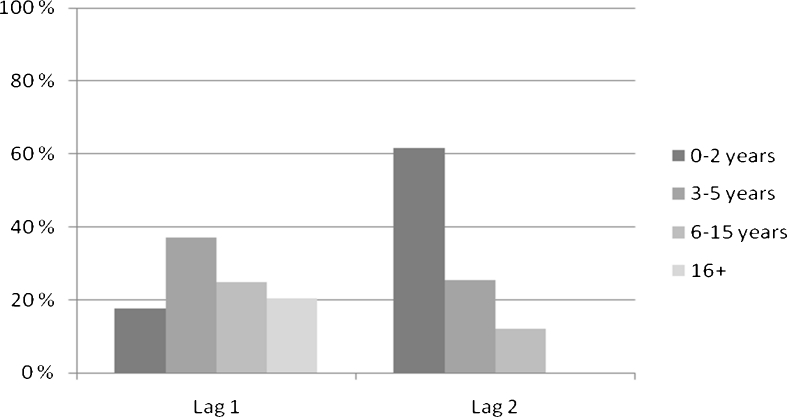
Lag-times. Lag 1 is the time between 1st gambling experience and active gambling. Lag 2 is the time between active gambling and gambling problem

The subjects started active gambling (gambling at least 3 times per week) usually before the age of 30. However, there was a significant gender-difference in the initiation ages: men started active gambling significantly earlier than women (*β* = −0.56, se = 0.23, *p* = 0.02) (Table 2). Other basic variables (education, marital status, residence, smoking, or debts) were not significantly associated with the age of active gambling. Lag-time between the first gambling experience and active gambling varied greatly. In contrast, active gambling very rapidly led to gambling problems. Within 2 years of active gambling, 62 % of the subjects reported having such a serious problem with the gambling, that they started to seek help (Fig. 2).

According to SOGS, the most popular games were slot machines (Table 4). Men tended to gamble more with sports and race betting, whereas weekly lotteries and scratch cards were preferred by women.

**Table 4:** Games gambled by the subjects more than once a week (according to SOGS). The number of respondents (female:male) and the relative % is described separately for both sexes (female *n* = 8, male *n* = 31)

	n (female:male)	% of females	% of males	% of all
Slot machines	6:23	75 %	74 %	74 %
Scratch cards	2:4	25 %	13 %	15 %
Sports betting	1:21	13 %	55 %	56 %
Horse races	0:3	0 %	10 %	8 %
Casino games at Finnish casino	0:4	0 %	10 %	10 %
Casino games elsewhere	0:4	0 %	10 %	10 %
Weekly lotteries	4:13	50 %	42 %	44 %
Daily lotteries	3:8	38 %	26 %	28 %
Veikkaus lottery tickets from internet	0:4	0 %	10 %	10 %
PAF^a^ Internet poker	1:9	13 %	29 %	26 %
PAF^a^ online games (except poker)	2:7	25 %	23 %	23 %
International Internet netpoker	0:7	0 %	23 %	18 %
International online games (except poker)	1:6	13 %	19 %	18 %
Private gambling	0:3	0 %	10 %	8 %
Speculation with investments or shares	0:1	0 %	3 %	3 %

^a^PAF is an internet gambling provider in the Finnish Ahvenanmaa Islands

## Discussion

One of the most interesting findings of this study was that pathological gambling behavior developed very rapidly after active gambling was initiated. Gambling that disturbed their life so much that they started to seek help to quit gambling was reported by 62 % of the subjects to have begun within 2 years of the onset of active gambling. In the case of drug addiction, the time from initiation to developing an addiction differs with different drugs: drugs that produce addiction more rapidly are generally considered to be more addictive. The development of addiction depends often upon the amount of reinforcement arising from the addictive behavior and how soon after the behavior the reinforcement occurs. Usually addictions develop slowly over years and decades (Vaillant 1998). However, certain drugs, e.g., heroin, cocaine and nicotine, can bring about addiction in the user very rapidly, probably because they produce a stronger reinforcement. Similar to certain drugs, gambling also seems able to cause strong and rapid reinforcement, which may lead to a rapid development of addiction.

Another interesting finding of this study was the gender-specific difference in the initiation ages. Men started active gambling (gambling at least 3 times per week) significantly earlier than women (*p* = 0.02). Our results are in line with previous findings (Granero et al. 2009; Crisp et al. 2004; Ladd and Petry 2002; Grant and Kim 2002; Tavares et al. 2001). Since early initiation to gambling is a known risk factor for PG (Kessler et al. 2008; Lynch et al. 2004), boys seem to be at higher risk of developing PG. This should be taken into account in the planning of prevention programs for youth.

The higher the subjects scored on SOGS, the more often they had gambling debts (*p* = 0.02). To fund their gambling, subjects borrowed money from various sources (Table 1). According to our results, the subjects borrowed money from banks and other external sources rather than from their spouses or relatives. This observation suggests that the subjects tried to hide their problem from significant others. Similarly, according to SOGS, 84 % of the subjects were hiding gambling from significant others, and the answers in the DSM-IV screen confirmed this: 92 % of the subjects reported telling lies and hiding gambling losses. Earlier studies have also reported that gambling often causes conflicts inside families (Johansson et al. 2009). This is in agreement with our findings: 95 % of the subjects reported in SOGS that they often have conflicts with significant others about gambling.

Pathological gamblers have often made many unsuccessful attempts to quit gambling before seeking treatment for the problem. This phenomenon was also demonstrated in our study: the thoughts of the subjects were preoccupied with gambling (97 %), and they felt restless or irritable when attempting to cut down or quit gambling (97 %). Most of the subjects (97 %) had made unsuccessful attempts to try to control or stop gambling. Thus in many ways gambling seems to resemble other addictions in how it affects the life and behavior of the addicted person: the PG’s have withdrawal symptoms, try to hide their behavior, experience feelings of guilt and shame, and even borrow money to continue the injurious behavior. These symptoms are similar to those used in the diagnosis of substance use disorders. However, PG not only resembles substance use disorders, it often co-occurs with them (Park et al. 2009; McGrath and Barrett 2009).

According to our results both men and women used slot machines weekly but certain sex-specific differences were also seen: men preferred sports betting, the races, and casinos more than women did (Table 4). On the other hand, women gambled more with scratch cards, and entered more weekly and daily lotteries than men did. Most studies suggest that women prefer non-strategic games, such as slot machines, whereas men tend to choose strategic games, such as sports betting and horse races (Granero et al. 2009; Grant and Kim 2002). Our results partly support these earlier observations: the men had a higher tendency towards strategic games whereas the women preferred luck-based, nonstrategic games. However, the most popular games of both sexes were slot machines. Men were more active in gambling through the internet (Table 4). Also earlier studies have reported that internet gamblers are more likely to be young men (Griffiths et al. 2009).

This study has several limitations. First, the sample size was relatively small: only 39 subjects. Second, the patients were recruited by announcements, and this may have caused bias among the study subjects. It has been suggested that older PG’s do not seek help as often as younger PG’s (Grant et al. 2009). This may, in part, explain why most of our subjects (54 %) were fairly young: under the age of 35. Third, in addition to the age bias, there was a gender bias, with four times as many men in the study. The uneven age and gender ratios probably affected the results, thus limiting the strength of the interpretations. The findings of this study need replication and extension among larger groups of treatment-seeking gamblers.
